# Does the Upstream Region Possessing MULE-Like Sequence in Rice Upregulate *PsbS1* Gene Expression?

**DOI:** 10.1371/journal.pone.0102742

**Published:** 2014-09-26

**Authors:** Mohammed Nuruzzaman, Tatsuo Kanno, Rika Amada, Yoshiki Habu, Ichiro Kasajima, Toshiki Ishikawa, Maki Kawai-Yamada, Hirofumi Uchimiya

**Affiliations:** 1 Institute for Environmental Science and Technology, Saitama University, Saitama city, Saitama, Japan; 2 Japanese Science and Technology Agency, PRESTO, Saitama, Japan; 3 Plant Genome Engineering Research Unit, Agrogenomics Research Center, National Institute of Agrobiological Sciences, Ibaraki, Japan; 4 National Agriculture and Food Research Organization, Institute of Floricultural Science, Tsukuba, Japan; 5 Graduate School of Science and Engineering, Saitama University, Saitama City, Saitama, Japan; Ben-Gurion University, Israel

## Abstract

The genomic nucleotide sequences of *japonica* rice (Sasanishiki and Nipponbare) contained about 2.7-kb unique region at the point of 0.4-kb upstream of the *OsPsbS1* gene. In this study, we found that *japonica* rice with a few exceptions possessing such DNA sequences [denoted to *Os*MULE-*japonica* specific sequence (JSS)] is distinct by the presence of *Mutator*-like-element (MULE). Such sequence was absent in most of *indica* cultivars and *Oryza glaberrima*. In *Os*MULE-JSS1, we noted the presence of possible target site duplication (TSD; CTTTTCCAG) and about 80-bp terminal inverted repeat (TIR) near TSD. We also found the enhancement of*OsPsbS1* mRNA accumulation by intensified light, which was not associated with the DNA methylation status in *Os*MULE/JSS. In addition, *O*. *rufipogon*, possible ancestor of modern rice cultivars was found to compose *PsbS* gene of either *japonica* (minor) or *indica* (major) type. Transient gene expression assay showed that the *japonica* type promoter elevated a reporter gene activity than *indica* type.

## Introduction

Non-photochemical quenching (NPQ) regulates energy conversion in photosystem II, thereby protecting plants from photoinhibition. Light essential for photosynthesis could cause damages of the photosynthetic apparatus. Under excess light, plants dissipate unused light energy in the antenna proteins of PSII. This mechanism is called energy-dependent thermal dissipation, which can be measured as a component of NPQ. Photoinhibition induced by high illumination results in photo damage with inactivation of PSII. Thus NPQ is considered as a good indicator for photoinhibition. We observed the NPQ capacity in a number of rice cultivars; NPQ was triggered by high light intensities [1]. Photoinhibition is caused by high light stress: excessive energy results in photodamage with inactivation of the PSII machinery. As a consequence, a decrease in the photochemical rate constant and thermal loss of energy is triggered by photoinhibition through inactivation of a part of the PSII reaction center. In our previous report, NPQ capacity was linked to the gene, *OsPsbS1* encoding PsbS protein [1]. Notably, *japonica* cultivars possessed an additional 2.7-kb region at the point 0.4-kb upstream of the *OsPsbS1*. The results of NPQ measurement in rice core collections concluded that NPQ capacity is generally higher in some *japonica* than in *indica*. On the contrary, with a few exceptions, there was no difference in terms of NPQs between subgroups within *indica* (aus and *indica*) or within some *japonica* subgroups (temperate *japonica* and tropical *japonica*) [1].

Previously, we postulated that the mutation regulated the difference in NPQ between *indica* and *japonica* [1]. In this work, we found that the mutation was due to the insertion of a *Mutator*-like element (MULE). *Mutator* (*Mu*) and *Mutator*-like elements (MULEs) are DNA transposons capable of moving from one genomic location to another by increasing their copy numbers. Using *Mu* transposon of maize, a number of reports for gene identification and mutagenesis are available [2]. The *Mu* transposable elements are a major class of class II transposons that was first discovered by Robertson in maize [3]. *Mu* and MULEs are distinguishable from other DNA transposable elements by having a 9 to 11-bp target site duplication (TSD), which flanks the element and are formed during transposition into a new genomic location. The terminal inverted repeats (denoted to TIRs) of MULEs, typically ranging from 100 to 500-bp, appear to be critical for element transposition and expression. *Mu* TIRs contain binding sites for the transposase where MURA protein was shown to bind a conserved ∼32-bp sequence motif in active *Mu* elements in maize [4]. As an essential structural component of the element, TIR plays important roles in transposition. In maize, the *Mu* system is composed of a number of different variants, all having 200-bp terminal inverted repeats, but each containing unrelated internal sequences [5], [6]. Computational analyses of rice genomic sequence indicate that, despite its small size, over 40% is repetitive DNA related to transposable elements [7]. A typical *Mu* insertion is accompanied by the duplication of 9-bp flanking sequence TSD; *Mu* exhibits a preference for insertion in genes possessing functions [8].

Here, we report that the *OsPsbS1*promoter sequences containing MULE like sequence promote the gene regulating non-photochemical quenching in rice.

## Materials and Methods

### Plant materials

All plant materials [e.g., *Oryza rufipogon* [perennial (P), annual (A) types, and accessions], *O*. *glaberrima*, *O*. *barthii*, *O*. *meridionalis*, *O*. *glumaepatula*, *O*. *officinalis*, *O*. *minuta*, *O*. *alta*, *O*. *punctata*, and *O*. *eichingeri*, and *O*. *sativa* cultivar Nipponbare, Sasanishiki, and Habtaki)] were kindly provided by the Laboratory of Food and Biomolecular Science, Graduate School of Agricultural Science, Tohoku University, Sendai, Japan and the Gene Bank of the National Institute of Agrobiological Sciences (NIAS). Classification of cultivars into *indica*, *temperate japonica*, and *tropical japonica* groups was described by Kojima et al. [9]. The classification has been updated with additional genetic information from NIAS (https://www.gene.affrc.go.jp/databases-core_collections_wr.php). All plants were grown in a greenhouse at 28°C (day) and 24°C (night). The maximum mid-day light intensity [photosynthetic photon flux density (PPFD)] reached to 1,000 µmol m^−2^ s^−1^ under this condition.

### Identification of MULE-like elements

The sequence *PsbS1* gene for rice [*Oryza sativa* (*Os*) ssp. *japonica* cv. Nipponbare] pseudomolecules was downloaded from the rice annotation group at Michigan State University (MSU, http://rice.plantbiology.msu.edu/, release 6.0). To search for putative autonomous MULEs in the rice genome, the corresponding regions of *OsPsbS1* gene sequence (*japonica*) (accession No AP003286.3) were used as queries to search against rice genomic sequences with tblastn program at the National Center for Biotechnology Information (http://www.ncbi.nlm.nih.gov). Multiple sequence alignment was performed by the software CLUSTAL W (http://www.ebi.ac.uk/clustalw) for sequence similarity and length. To determine the abundance of MULE-like elements in rice genome, copy number was estimated by considering one pair of TIRs as one element. To estimate how many elements are associated with a TSD, the presence of a TSD was verified using a pipeline consisting of *japonica*, *indica*, and *O*. *glaberrima* genome that search for 9 to 12-bp direct repeat with no more than 3 mismatches flanking the ends of the TIR sequences. The copy number of autonomous MULE elements was estimated from all elements retrieved from the previous step having significant match to known MULE transposases (*E* = 10^−5^, BLASTX) after filtering for low complexity. To search for MULEs with left TIR, elements from each genome sequence were identified using BLASTN program for *japonica* (http://rapdb.dna.affrc.go.jp/tools/blast) and for *indica* and *glaberrima* (http://www.gramene.org/Multi/blast). The following criteria were used to filter the results: TIRs must be at least 60-bp long, [10] truncations at the external ends of TIRs must be no more than 12-bp, [11] the two TIRs on one or both ends are less than 200-bp apart, and presence of a 9 to 12-bp TSD with no more than 3 mismatches [2]. The following criteria were used to define element having multiple copies in a genome. For individual elements, if the TIRs of two elements (with different TSDs) can be aligned (BLASTN, *E* = 10^10^), and if>95% of the sequence between the TIRs can be aligned (BLASTN, *E* = 10^−10^), then the two elements are defined as copies. The elements were located on rice chromosomes according to the positions specified in the MSU rice database.

### Quantitative measurement of transcripts

Plants were grown in a greenhouse at 28°C under 12 light (PPFD, ca. 240 µmol m^−2^ s^−1^) and 12 h dark conditions (defined as “low light” condition). High light condition (PPFD, ca. 430 µmol m^−2^ s^−1^) was made by using an additional high pressure metal halide lamps. RNA was extracted from the mixture of third to fifth leaves by PureLink RNA mini kit (Life Technologies) according to the manufacturer's protocol. cDNA was synthesized using Transcriptor First Strand cDNA Synthesis kit (Roche) according to the manufacturer's instruction using oligo d(T)18 primer and one microgram of DNase treated total RNA. Real time PCR was carried out by SYBR Premix Ex Taq II (TAKARA) according to the manufacturer's protocol using one microliter of cDNA as a template. The data were collected by Thermal Cycler Dice (Takara), and analyzed with Thermal Cycler Dice Real Time System Single Ver. 4.02 software. The relative expression of each sample was normalized by *Actin* mRNA. Primers are listed in Table S1. Statistical analysis was performed for analysis of variance (ANOVA) followed by the Fisher's least significant difference multiple-comparison test.

### Bisulfite genomic sequencing for methylation assay

Genomic DNA was extracted from young leaves with DNA Plant Easy kit (QIAGEN) and bisulfite treatment was performed with MethylCode Bisulfite Conversion Kit (Life Technologies). PCR amplification was done with Advantage 2 polymerase (Clonetech), and cloning and sequencing of amplified fragments were done as described [12]. Conversion rates in the bisulfite reactions were evaluated for the region in the mitochondria genome (Sasanishiki 97.7%, Habataki 98.6%). Primers used in this study are listed in Table S1.

### Measurement of chlorophyll fluorescence

Third leaves of 3-week-old plants or fourth leaves of 4-week-old plants were analyzed. Excised leaves (∼0.5 cm^2^) were floated on ion exchanged water containing 0.01% Triton X-100 or 0.1% agar in 24-well plastic plates. Samples were predark-adapted in room (PPFD <10 µmol m^−2^ s^−1^) for at least 2 h. Chlorophyll fluorescence was measured with a Closed FluorCam (Photon Systems Instruments; [1]. Samples were dark adapted for 5 min before Fo and Fm were measured. Actinic lights were supplemented for 5 min before measurements of Fm' to calculate parameter NPQ. NPQ value represents the size of NPQ rate constant relative to size of the basal dissipation's rate constant, calculated by Stern–Volmer equation on nonphotochemical processes [13]. Formulas used here for ΦNPQ and ΦNO are the alternatives [14] to the original notations [15].

### Vector construction and transient GUS-reporter assay

The promoter fragments of *OsPsbS1* genes were amplified from genomic DNA of *japonica* (Sasanishiki) and *indica* (Habataki) by PCR with a common primer set (5′-CACCACCTGGAGTTAGTACACAGCACATACTGAC-3′ and 5′-GGCTCCCGACACCAGCATCGACTGCGCCAT-3′). The promoter region consisted of 4581 bp of the *japonica* and 1905 bp of the *indicia* promoter regions and the N-terminal 10 amino acids of *OsPsbS*. These fragments were subcloned into the pENTR/D-TOPO vector (Invitrogen) and then integrated into a Gateway destination binary vector pMDC164 [16], resulting in the promoter-β-glucuronidase (GUS) reporter plasmid consisted of each of the *OsPsbS1* promoter region and the GUS reporter for transformation of plants. The constructs were transformed into the *japonica* (Sasanishiki) or *indica* (Habataki) rice calli by the *Agrobacterium* mediated transformation method [17]. This experiment was repeated four times. After infection, calli were co-cultured with *Agrobacterium* at dark, high light (PPFD, ca. 430 µmol m^−2^ s^−1^) and low light (PPFD, ca. 240 µmol m^−2^ s^−1^) for additional 3 days. Total protein extracted from calli in the extraction buffer (0.1 M NaHPO_4_ buffer pH 7.0, 10 mM EDTA, 0.3% sarcosyl (v/v), and 0.1% (v/v) TritonX-100) was used for GUS assay as described by Yoshizumi et al. [18]. A 50-µl aliquot of an extract containing 20 µg proteins was mixed with an equal volume of 1 mM 4-methylumbelliferyl-β-D-glucuronide in the extraction buffer and incubated for 2 h at 37°C. A 50-µl aliquot of each reaction was mixed with 1 mL of 0.2 M sodium carbonate, and the fluorescence (excitation at 365 nm and emission at 455 nm) was measured by a F-4500 fluorescence spectrophotometer (Hitachi).

### PCR amplification to detect 2.7-kb deletion in different rice species

Total DNA was extracted from leaves of all the wild rice species and cultivated rice Nipponbare, Sasanishiki, and Habataki with plant genomic DNA extraction mini kit. DNA was PCR-amplified with KOD Plus Neo (Toyobo) with primers PsbS1- ZF1 (5′- GCGAATGGCTGGATTTCGGGA -3′) and PsbS1-ZR1 (5′- CTTGGACCTGCCGAACAGCG -3′) for 40 cycles. Each PCR was performed (repeated three times) in an ABI 9700 thermocycler (Applied Biosystems) consisted of incubation at 94°C for 2 minutes, at 98°C for 10 s, at 68°C for 30 s, and at 68°C for 3 minutes. Other conditions followed the standard condition for three step reaction.

## Results

### MULE –like-sequence in the upstream region of *PsbS1* gene

**Figure 1:**
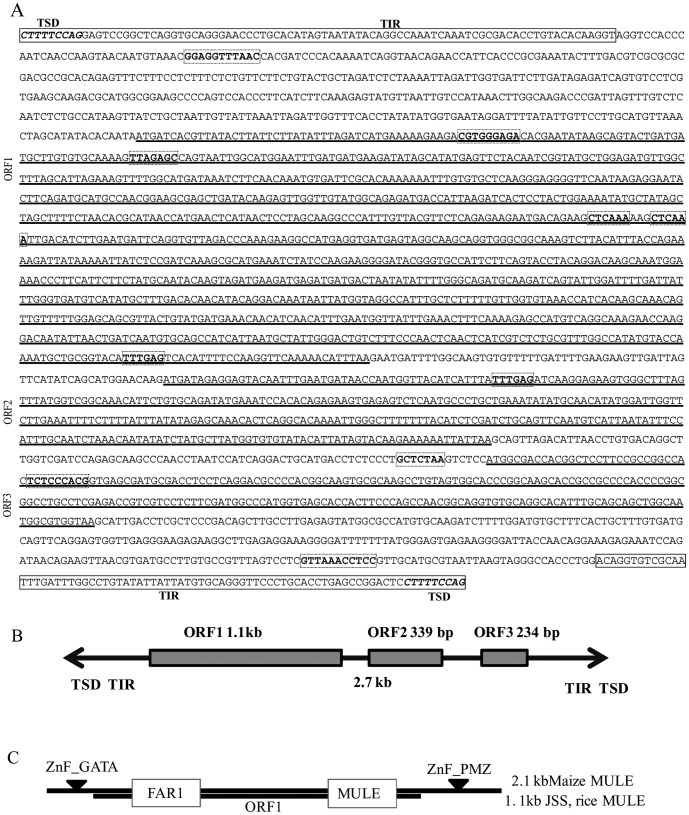
(A) Japonica specific sequence (JSS) terminal inverted repeat sequence (TIR) indicated by rectangle block and terminal target site duplication (TSD) indicated by *italic* in rectangle. Internal target side duplications are indicated by dot rectangle. Three open reading frames (ORFs) are underlined. (B) Japonica specific sequence (JSS) is around 2.7-kb, TIR indicated by arrow. Three open reading frames (ORFs) indicated by the rectangle. (C) Two domains (FAR1 and MULE) in the long open reading frame (1.1-kb) in JSS. FAR-RED IMPAIRED RESPONSE1 (FAR1) and Maize MULE had additional domains (ZnF_GATA = GATA zinc finger; ZnF_PMZ =  Plant mutator transposase zinc finger) FAR1 and MULE position indicated by white boxes.

Through the search of additional sequences upstream of *PsbS1* gene, we found conserved target site repeats in the *japonica* rice (JSS; *japonica* specific sequence). Regarding a typical MULE characteristics, we found the presence of 80-bp terminal inverted sequences on the opposite ends (alignment by CLUSTALW) of *Oryza sativa* (*Os*) *Os*MULE/JSS in *japonica* rice, cv. Nipponbare (Figure S1A). The *Mu* and MULEs were distinguishable from other DNA transposable elements by having a 9 to 11-bp TSD that most likely formed during transposition into a new genome location. In facts, there are 9-bp (CTTTTCCAG) TSD at both ends of the *Os*MULE/JSS (Figure 1A). The possible size of the *Os*MULE would be 2684-bp with an 80-bp TIR. One long open reading frame (ORF, 1.1-kb) and two small ORFs (339-bp and 234-bp) were found in the *Os*MULE/JSS sequence (Figure 1A and B). In the *Os*MULE/JSS, there are five internal inverted repeat (IIR) sequence varies 6-bp to 9-bp and formed ten DNA loops, whose lengths are not identical (Figure 1A and Figure S1B). We also observed FAR-RED IMPAIRED RESPONSE1 (FAR1) and MULE domains in the long ORF of JSS. FAR1 and MULE domains in this case were similar to part of maize MULE at the amino acids level, whereas maize MULE had two additional domains (e.g., ZnF-GATA and ZnF-PMZ; Figure 1C). Protein sequence of the longest ORF in rice is about 50% analogous to that of maize (MLOC100280229), confirmed by BLAST search in NCBI.

### Genome organization *Os*MULE in *japonica* and *indica* rice

**Figure 2:**
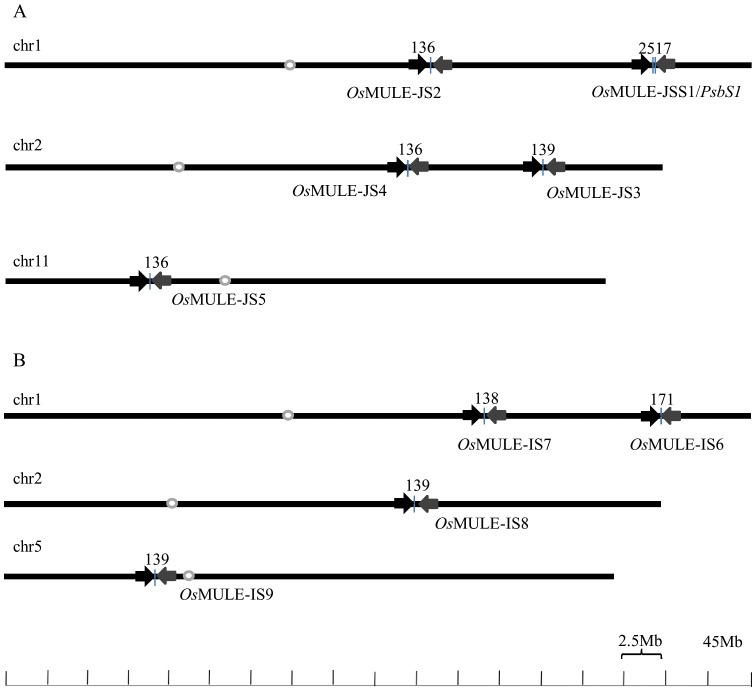
Structure and location of multiple copy number of terminal inverted repeat (TIR) sequence (80-bp) in different chromosome in (A) *japonica* cv. Nipponbare and (B) *indica* cv. 93-11. Different directions of TIRs were showed by arrow. The region (37694045–37696713) of chromosome 1 indicates *PsbS1* genes [1]. Chromosome numbers are indicated at the left side of each bar. The scale is in million bases (Mb). White dot on each chromosome shows the rough position of the centromere.

Through homology searches using BLASTN program with respects to 80-bp TIR sequences, 4 copies of TIR in chromosome (chr) 1 and chr 2, and 1 copy in chr 11 were counted in *japonica* cultivars (Figure 2A). Similarly, 2 copies of TIR in chr 1, and 1 copy in chr 2 and chr 5, respectively in *indica* rice cultivar (93–11) were presented (Figure 2B). All nine *Os*MULE with highly homologous TIRs existed in *japonica* (Nipponbare) and in *indica* (93–11) (Table S2). In *japonica*, one was found in the PAC clone P0677H08 (AP003286), which has been assigned to rice on chromosome 1. This PAC clone contained a sequence that was almost identical to JSS and its flanking sequence. Out of the 9 *Os*MULE elements, 5 (e.g., *Os*MULE-JSS1 and *Os*MULE-JS3) are located in the long arms of the relevant chromosomes, whereas 2 elements of 9 (e.g., *Os*MULE-JS5 and *Os*MULE-IS9) are located in the short arms of the relevant chromosomes (Figure 2 A and B). Eight elements were flanked by perfect 9-bp and 11-bp TSD, one element was flanked by imperfect 12-bp TSD (Table S2). Identity of left and right TIR was more than 95%.

### Transcript accumulation in different subspecies of rice

**Figure 3:**
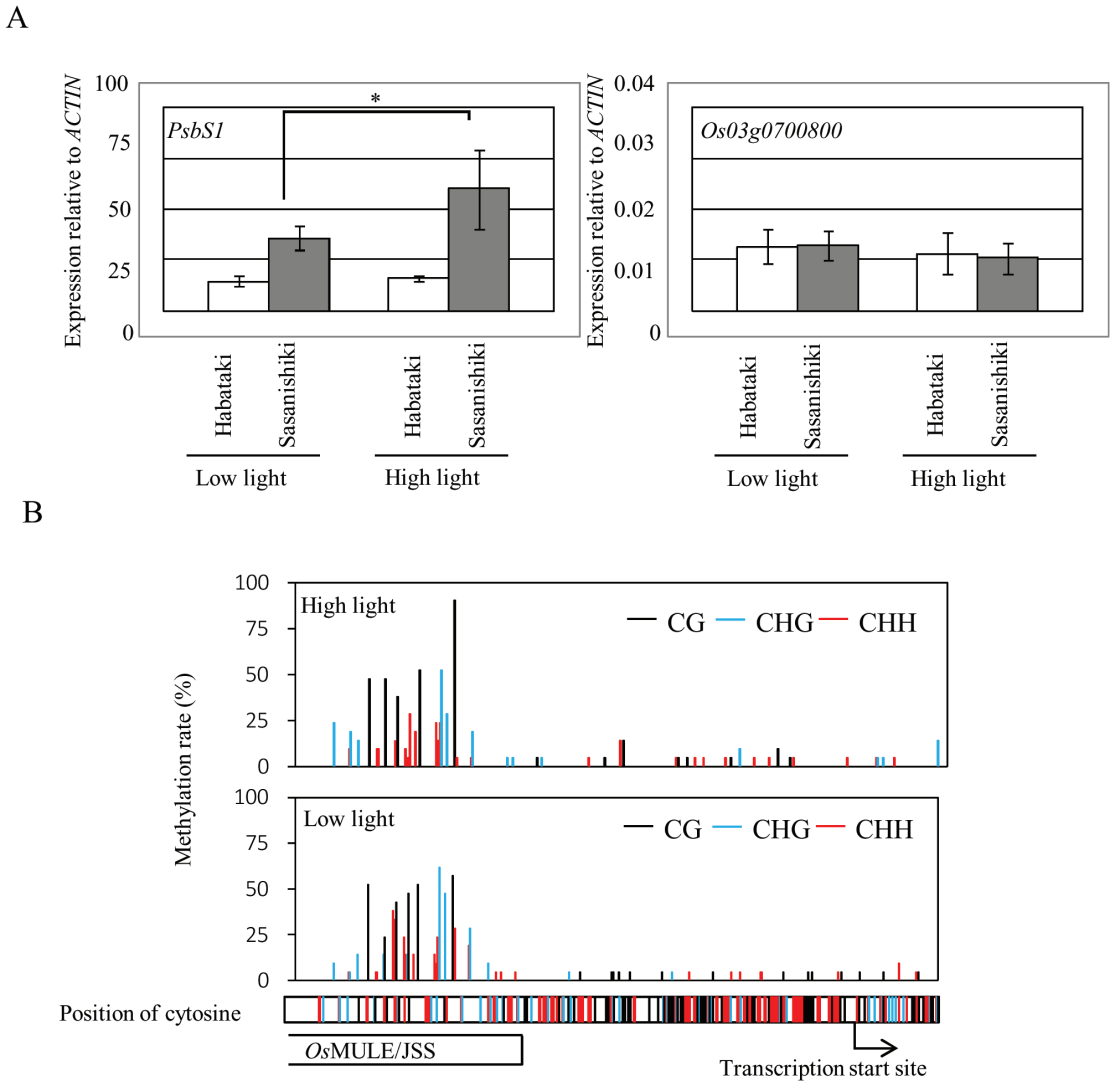
The effect of light condition to the *OsPsbS1* mRNA accumulation level and to the DNA methylation status at the 5′ upstream region of the gene. (A) The relative RNA accumulation level of *OsPsbS1* against *Actin* mRNA in two different rice cultivars (Habataki and Sasanishiki) under high (PPFD, ca. 430 µmol m^−2^ s^−1^) or low (PPFD, ca. 240 µmol m^−2^ s^−1^) light conditions. The accumulation of *Os03g0700800* mRNA was shown as an internal negative control to the light conditions. Error bars indicate the standard error among three biological replicas. Asterisk indicates significant differences by Fisher's least significant difference multiple-comparison test (*P*<0.01). (B) DNA methylation status at the 5′ upstream region of *OsPsbS1* in cultivar Sasanishiki under high (upper panel) or low (middle panel) light conditions. All the position of cytosine in the sequenced region (about 0.6 kb) was shown in the bottom panel. CG, CHG, and CHH methylation are indicated in black, blue and red bar, respectively. *Os*MULE/JSS region was shown in open box. The bent arrow indicates the transcription start site of the gene.

To know the effect of light intensity on *OsPsbS1* mRNA accumulation level, we performed quantitative RT-PCR. In Habataki, the light intensity was not affected on the accumulation level of *OsPsbS1* mRNA. In Sasanishiki, high light condition caused over 1.5–fold accumulation of *OsPsbS1* mRNA, relative to low light condition (Figure 3A). We subsequently carried out bisulfite sequencing to understand how light intensity affects the DNA methylation status in *Os*MULE/JSS (inserted in 5′ upstream region in Sasanishiki*OsPsbS1* locus). Higher levels of cytosine methylation were observed within the *Os*MULE/JSS in Sasanishiki, and difference in the light intensity did not induce apparent changes in the methylation levels (Figure 3B). In contrast, methylation of the proximal upstream region (about 0.3 kb) of *OsPsbS1* gene was kept at low levels regardless of the light intensity. No cluster of methylated cytosines was observed in the upstream region of *OsPsbS1* gene in Habataki where the *Os*MULE/JSS is absent (Figure S2). The results indicate that the presence of methylated *Os*MULE/JSS in the upstream region of *OsPsbS1* gene could be responsible for higher expression levels and/or light inducibility of *OsPsbS1* gene in Sasanishiki (Figure 3A).

### *Os*MULE/JSS in rice and evaluation of promoter activities

**Figure 4:**
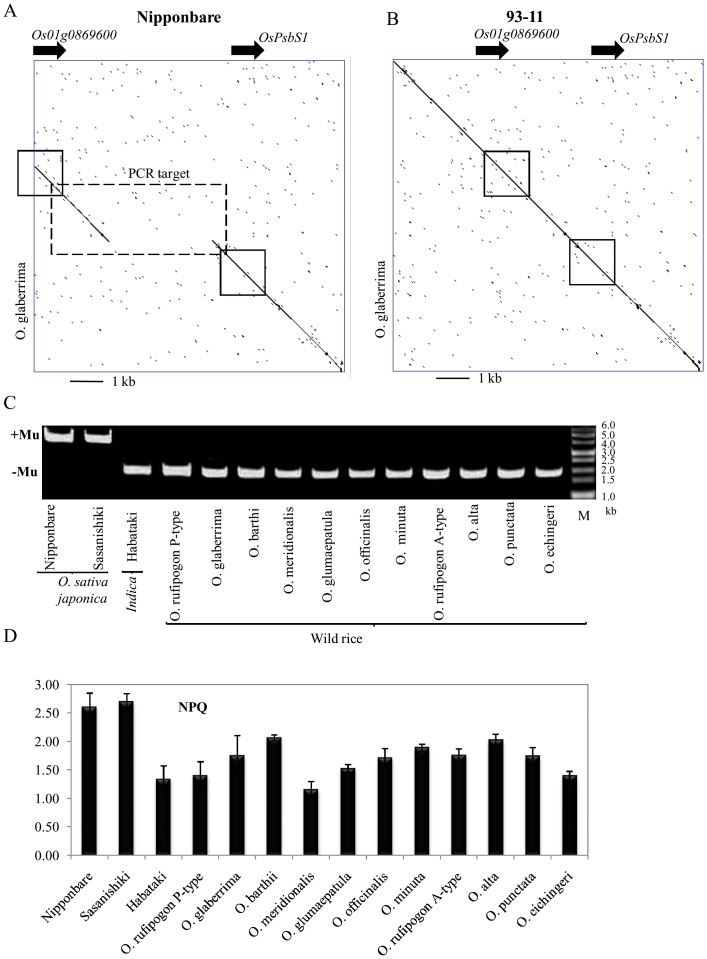
Comparison of PsbS genome sequence between *japonica* and *O. glaberrima*, and *indica* and *O. glaberrima*. (A) Genomic sequences of *japonica* cultivar (Nipponbare) and *O. glaberrima*, and (B) *Indica* cultivar (93–11) and *O. glaberrima* were compared by using a har-plot analysis (http://www.vivo.colostate.edu/molkit/dnadot) with a 9-base frame. Squares with solid lines represent genetic regions of *LOC-Os01g0869600* and *OsPsbS1* (Coding region). Rectangle with broken line in the box (Left) represents the target region of PCR. (C) PCR amplification of genomic sequence around the 2.7-kb deletion. *Mu*-like groups of the cultivars analyzed are indicated left side the gel image (+, *Mu* present; −, *Mu* absent). (D) Fully expanded third leaves of the all rice were used for NPQ measurement. Image of NPQ values under high illumination photosynthetic photon flux density (PPFD, 1,500 µmol m^−2^ s^−1^) for 5 min is shown. Data represent means and SDs.

**Figure 5:**
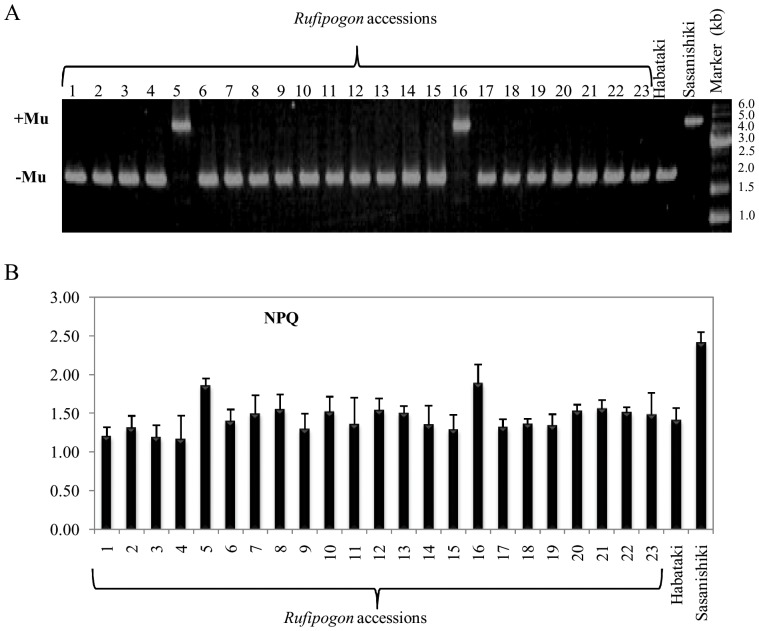
Comparison of PCR amplified products (A) and NPQ values (B) among the *rufipogon* accessions. For comparison, Habataki and Sasanishiki were used as control samples. Illustrated documents are same as in Figure 4.

In the previous study, we reported that most of *indica* cultivars do not possess the 2.7-kb region near the initiation site of translation of *OsPsbS1* gene [1]. Present study suggested that this 2.7-kb sequence did not result from deletion of genomic DNA, but insertion of transposon into *japonica* rice genome. Using *O*. *glaberrima* genomic sequence, we compared the nucleotide sequence around *OsPsbS1* between *japonica* cultivar (Nipponbare) and *O. glaberrima* (Figure 4A). The 2.7-kb region was lost in *O. glaberrima* in comparison to *japonica*; the other genomic sequence was identical (Figure 4A and B). To confirm such deletions, PCR analysis was performed on *japonica*, *indica* and several wild types of rice and *O. glaberrima* (Figure 4C). The *japonica* cultivars possessed 2.7-kb region, whereas Habataki (an *indica* cultivar) and wild rice species lacked the particular DNA (Figure 4C). We also compared chlorophyll fluorescence parameters among rice cultivars and wild rice species: Nipponbare and Sasanishiki (both *japonica* cultivar), Habataki (an *indica* cultivar), *O*. *glaberrima* and 12 wild rice species. Figure 4D shows the NPQ value during the transition from dark to high light conditions at a PPFD of 1,500 µmol m^−2^ s^−1^ for 5 min. Induction of NPQ in *japonica* rice leaves was dominated by a large transient rise during the 5 min of illumination. Habataki and all wild type plants had a NPQ lower than the *japonica* during this induction period (Figure 4D). Regarding the relationship between genomic sequence alteration and NPQ values, *japonica* cultivars expressed higher NPQ scores than *indica* cultivars and other rice species. In order to extend our knowledge on *OsPsbS* in *O*. *rufipogon*, we analyzed 23 accessions. As shown in Figure 5A, most accessions showed *indica* type DNA fragment by PCR, whereas two showed *japonica* type. This evidence was coincided with NPQ value (Figure 5B).

**Figure 6:**
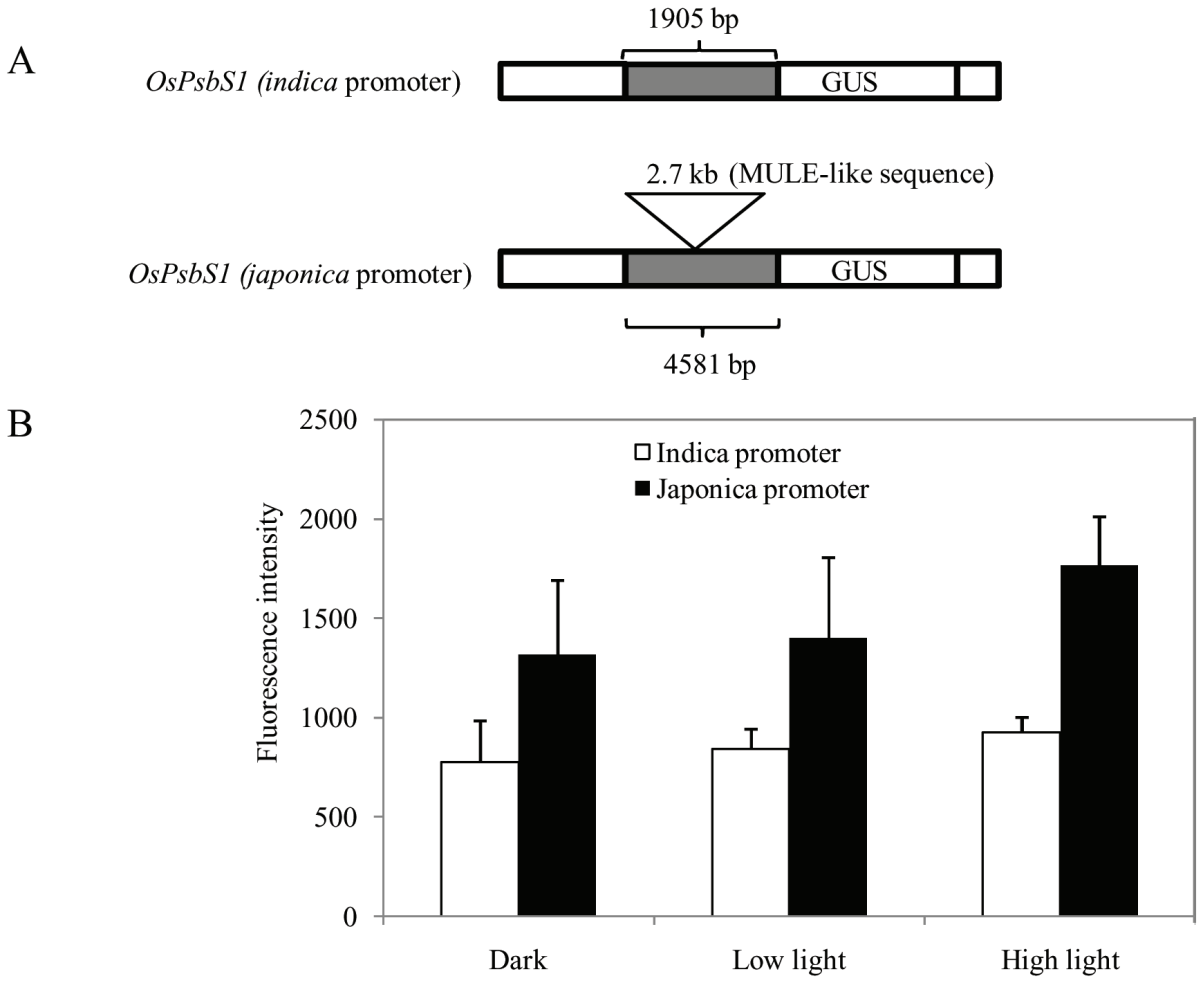
Illustrated presentation of a portion of binary vector pMDC164 possessing reporter gene and results of GUS analysis by transient expression of respective vectors. (A) A portion of vector with either *indica* promoter or *japonica* promoter is presented. (B) Comparison of GUS activity in calli treated with *Agrobacterium* possessing vector in “A”. Calli were cultured at dark or low light (PPFD, ca. 240 µmol m^−2^ s^−1^) or high light (PPFD, ca. 430 µmol m^−2^ s^−1^) for 3 days, followed by GUS measurements.

In order to evaluate promoter activities of *OsPsbS* genes between *indica* and *japonica* type, we constructed binary vector pMDC164 containing the promoter-β-glucuronidase (GUS), whose expression is directed by either *indica* or *japonica* type *OsPsbS* promoter (Figure 6). These constructs were transferred to rice calli (Sasanishiki or Habataki) by the *Agrobacterium* mediated method. After three days of incubation, GUS activities were measured. The Sasanishiki (*japonica*) type promoter showed higher GUS activities than *indica* type promoter (Figure 6).

## Discussion

We previously demonstrated that *japonica* cultivars possessed the 2.7-kb region at 0.4-kb upstream of the *OsPsbS1* gene; this region was absent in *indica* cultivars [1]. In this study, we found a new MULE-like element in *japonica* rice genome. MULEs play important roles in plant genome evolution due to their high activity and the potential to acquire and amplify gene fragments. In rice, at least 35% of genomic sequence is composed of residuals of transposons including hundreds of families of MULEs and Pack- MULEs (partial MULE sequences) [19], [20]. Some *gypsy*-type long-terminal repeat elements with high copy numbers were preferentially inserted into heterochromatic regions like centromeres [21]. Most of these elements were clustered in the gene-poor regions, presumably to avoid harmful effects of transposition on the host genome. Transposable elements are the major components of plant genomes and these elements survive and thrive in the genome through a variety of strategies. Most of the members of the *Os*MULE/JSS element are associated with distinct TSD (Table S2), suggesting that these elements are derived from transposition. The target sites of the *Os*MULE/JSS elements are very AT-rich (Table S2). *AtMu1*, the MULE that is active on the *ddm1* mutant background in *Arabidopsis*, and has similar target specificity for AT-rich sequence [22]. This is consistent with the fact that the catalytic domain of *Os*MULE/JSS is closely related to that of *AtMu1*. If the transposon preferentially inserts into AT-rich regions, it would be less likely for the element to insert into coding regions of genes that are relatively GC-rich. Such target site specificity may result from an efficient counter-selection effect of insertion within gene-rich region. The target site preference of the *Os*MULE/JSS is different from GC-rich *Mu* elements [23], [24]. The average GC content is 44% in rice genome and 53% in the coding region of genes.

All *Os*MULE are not only highly homologous 80-bp TIR but also a highly homologous internal sequence among these elements. Those are contained 9-bp target site duplication (CTTTTCCAG). For example, *Os*MULE-JS2 and *Os*MULE-IS6 showed homology (100%), on the basis of internal sequence similarity by the BLAST 2 sequence (http://redb.ncpgr.cn/index.php; REDB database). The internal regions of these elements were significantly homologous to *Os*MULE-JSS1 (Table S2). These sole-TIRs may belong to truncated or degenerated MULEs that share no internal sequence similarity with other members investigated. Recombination may be involved in generation of these elements. These results suggested that the elements (e.g., *Os*MULE-JS2 and *Os*MULE-IS7) originated from an active version of *Os*MULE-JSS1 or its paralogs via the interrupted gap-repair mechanisms. Besides the 9 family members, *Os*MULE-JSS1element does not have any closely related families in the other rice genome except *japonica* cultivar; the best matches at the nucleotide level are 1418-bp stretches in the most conserved transposase domain with a similarity of 53% in *O*. *minuta* and 31% in *O*. *glaberrima*, respectively, using NCBI nucleotide blast search. On the basis of this finding, we concluded that JSS and/or the *Os*MULEs found in different chromosomes represented the same sequence in the rice genome. Five elements (*Os*MULE-JSS1 to *Os*MULE-JS5) were found and had been assigned to chromosomes 1, 2, and 11 in *japonica*. Four elements (*Os*MULE-IS6 to *Os*MULE-IS9) were also assigned to chromosomes 1, 2, and 5 in *indica* (Table S2). All of them are newly identified by the present study.

This type of transposon is known to be hypomethylated [22]. In fact, metylation of a part of *Os*MULE-JSS1 was observed, regardless of the light intensity. Thus, mechanisms other than methylation are likely operative in transcriptional activation triggered by high light.

The TIR sequences of DNA elements contain *cis*-elements (e.g., TGCAGG, CACCTG, and GTAC; Figure 1A) that are responsible for recognition by the relevant transposases. It also contributes to the selection of insertion site, and is a target site for the epigenetic regulation [25], [26]. As a result, the TIR sequences play a critical role in the successful amplification of relevant transposon element.

Rice is believed to become domesticated ∼9,000 y ago [27]–[30]. We hypothesize that MULE transposon events in our target gene took place prior to the divergence time between tropical *japonica* and *indica* some ∼3,900 y ago [30]. For the evaluation of divergency of rice, following genes have been utilized:*sh4* that confers non shattering [31], [32], plant architecture *prog1* [33] loci, red pericarp *rc* [34], *BADH2* fragrance gene [35], the *sd1*semidwarfing gene [36], the *Pi-ta* disease resistance locus [36], the starch biosynthetic gene *Wx* [36], and the *GS3* grain length gene [37].

The present study showed that several wild species of rice and *O*. *glaberrima* expressed lower NPQ values than *japonica* rice (Figure 4). According to the reported phylogenic tree, *O*. *glaberrima* is distantly related to *indicia*, *japonica*, and other wild species of rice [38]–[40]. Thus emphasis was made in the comparison of such genome structure between *japonica* and *O*. *Glaberrima*. Surprisingly, *O*. *glaberrima* showed *indica* type NPQ response as well as genome structure in the neighboring sequences of the target gene. It was noteworthy to mention that most accessions of *rufipogon* possessed *indica* type *PsbS* genes, whereas two out of 23 showed *japonica* type *PsbS* genes (Figure 5). These results support the hypothesis that *rufipogon* is the purported ancestor of modern rice cultivars. Because the *rufipogon* is composed of heterogeneity in terms of the presence of *PsbS* gene of either *japonica* or *indica* type. This may explain that some rice contained different types of *OsPsbS1*gene promoter in either *indica* or *japonica*. Furthermore, it is important to assess the promoter activities to answer the question whether or not the *japonica* promoter possessing MULE-like sequence is responsible for the upregulation of the target gene. In this respect, we showed the direct evidence that *japonica* type promoter elevated GUS activities (reporter gene) than *indica* type (Figure 6). Detailed dissection of the MULE-like sequence to understand the precise mechanism of promoter activation and the knowledge of the evolution in rice will be a subject of the further studies.

## Supporting Information

Figure S1 **Structure of Japonica specific sequence (JSS).** (A) Left and right terminal inverted repeats of JSS are alignment by CLUSTALW. (B) Ten DNA loops formation in JSS and mechanism of transposition catalyzed by the transposase.
(PDF)

Figure S2 **Comparisons of the DNA methylation status at the 5′ upstream region of the** ***OsPsbS1*** **gene between Habataki and Sasanishiki.** All the position of cytosine in the sequenced region (about 0.6 kb for Sasanishiki and about 0.75 kb for Habataki) was shown under the methylation rate panel, respectively. CG, CHG, and CHH methylation are indicated in black, blue and red bar, respectively. *Os*MULE/JSS region was shown in open box for (A) Sasanishiki and for (B) Habataki, the *Os*MULE/JSS insertion site in Sasanishiki was pointed by an arrow. The bent arrow indicates the transcription start site of the gene.
(PDF)

Table S1 **The sequence of primers used for quantitative RT-PCR (qRT-PCR) amplification and for methylation analysis.**
(DOC)

Table S2 **Summary of characteristics of** ***Os*** **MULE elements in both** ***japonica*** **and** ***indica*** **rice cultivars.**
(DOC)
