# Supplementary material for: Does the Upstream Region Possessing MULE-Like Sequence in Rice Upregulate PsbS1 Gene Expression?
Source: PLoS One. 2014 Sep 26;9(9):e102742. doi: 10.1371/journal.pone.0102742 (PMC4178011; doi:10.1371/journal.pone.0102742)
Supplement: Figure S1 — Structure of Japonica specific sequence (JSS). (A) Left and right terminal inverted repeats of JSS are alignment by CLUSTALW. (B) Ten DNA loops formation in JSS and mechanism of transposition catalyzed by the transposase. (PDF) [file pone.0102742.s001.pdf]

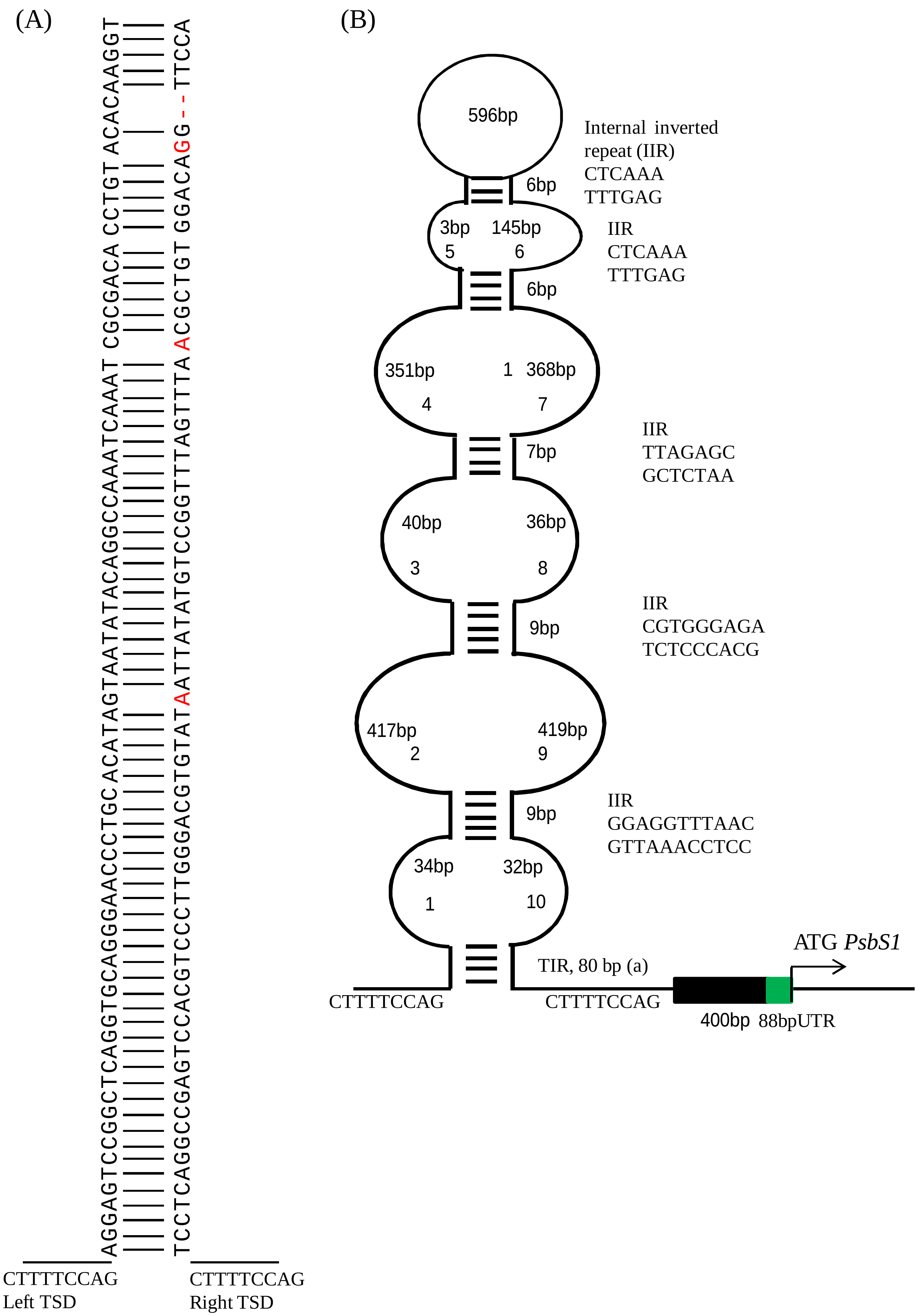

(A)
AGGAGTCCGGCTCAGGTGCAGGGAACCCTGCACATAGTAATATATACAGGCCAAATCAAAT CGCGACA CCTGT ACACAAGGT
TCCTCAGGCCGAGTCCACGTCCCTTGGGACGTGTATAATTATATGTCCGGTTTAGTTTA ACGCTGT GGACAGG - - TTCCA
CTTTTCCAG
Left TSD
CTTTTCCAG
Right TSD
(B)
596bp
Internal inverted repeat (IIR)
CTCAAA
TTTGAG
6bp
3bp
145bp
5
6
IIR
CTCAAA
TTTGAG
6bp
351bp
1
368bp
4
7
IIR
TTAGAGC
GCTCTAA
7bp
40bp
36bp
3
8
IIR
CGTGGGAGA
TCTCCCACG
9bp
417bp
2
419bp
9
IIR
GGAGGTTTAAC
GTTAAACCTCC
9bp
34bp
32bp
1
10
TIR, 80 bp (a)
ATG PsbS1
CTTTTCCAG
CTTTTCCAG
400bp
88bpUTR

Figure S1. Structure of Japonica specific sequence (JSS) (A) Left and right terminal inverted repeats of JSS are alignment by CLUSTALW. (B) Ten DNA loops formation in JSS and mechanism of transposition catalyzed by the transposase.
