# Supplementary material for: Does the Upstream Region Possessing MULE-Like Sequence in Rice Upregulate PsbS1 Gene Expression?
Source: PLoS One. 2014 Sep 26;9(9):e102742. doi: 10.1371/journal.pone.0102742 (PMC4178011; doi:10.1371/journal.pone.0102742)
Supplement: Figure S2 — Comparisons of the DNA methylation status at the 5′ upstream region of the OsPsbS1 gene between Habataki and Sasanishiki. All the position of cytosine in the sequenced region (about 0.6 kb for Sasanishiki and about 0.75 kb for Habataki) was shown under the methylation rate panel, respectively. CG, CHG, and CHH methylation are indicated in black, blue and red bar, respectively. OsMULE/JSS region was shown in open box for (A) Sasanishiki and for (B) Habataki, the OsMULE/JSS insertion site in Sasanishiki was pointed by an arrow. The bent arrow indicates the transcription start site of the gene. (PDF) [file pone.0102742.s002.pdf]

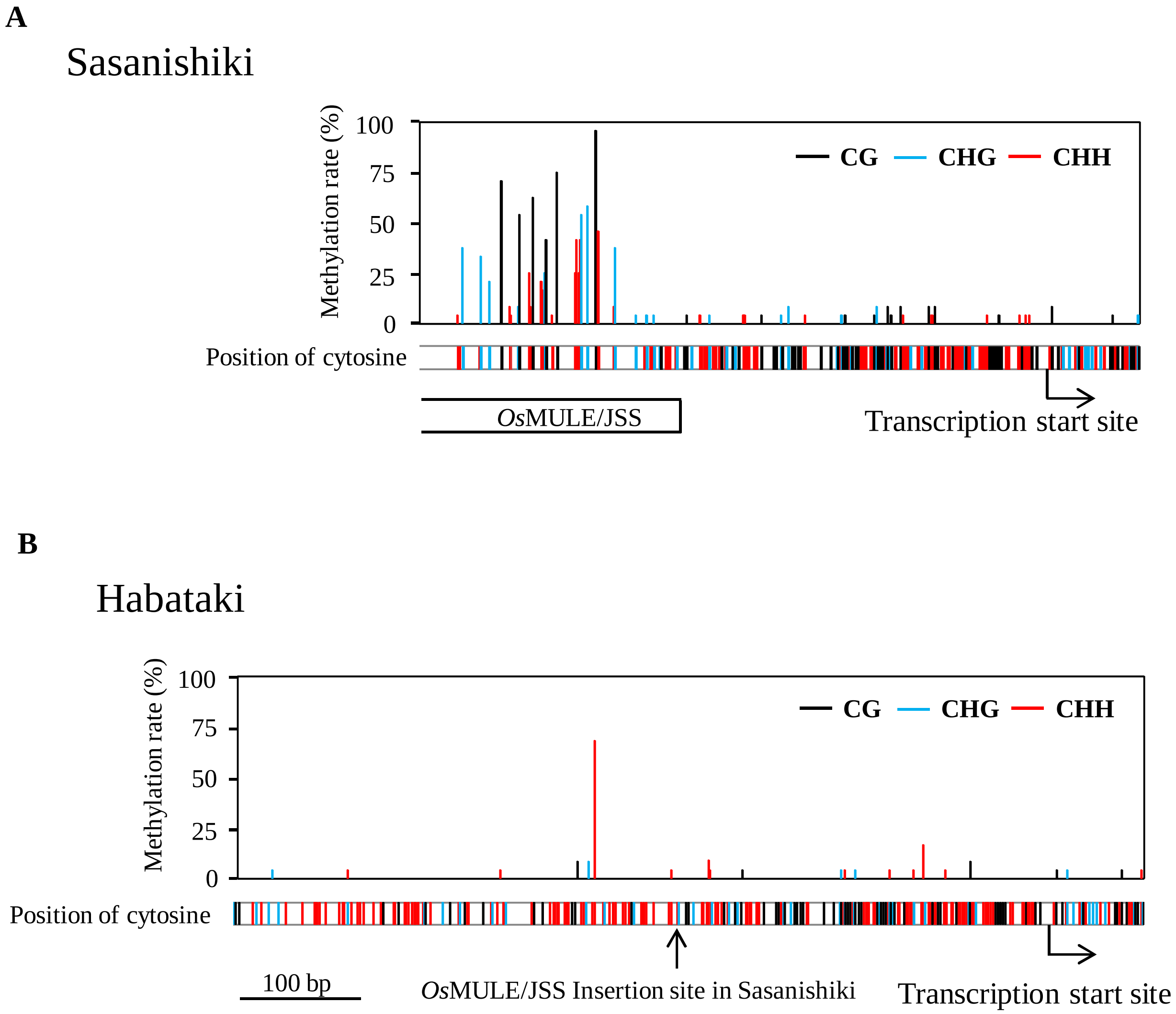

Figure S2. Comparisons of the DNA methylation status at the 5' upstream region of the *OsPsbS1* gene between Habataki and Sasanishiki. All the position of cytosine in the sequenced region (about 0.6 kb for Sasanishiki and about 0.75 kb for Habataki) was shown under the methylation rate panel, respectively. CG, CHG, and CHH methylation are indicated in black, blue and red bar, respectively. *Os*MULE/JSS region was shown in open box for (A) Sasanishiki and for (B) Habataki, the *Os*MULE/JSS insertion site in Sasanishiki was pointed by an arrow. The bent arrow indicates the transcription start site of the gene.
