# Supplementary material for: Does the Upstream Region Possessing MULE-Like Sequence in Rice Upregulate PsbS1 Gene Expression?
Source: PLoS One. 2014 Sep 26;9(9):e102742. doi: 10.1371/journal.pone.0102742 (PMC4178011; doi:10.1371/journal.pone.0102742)
Supplement: Table S1 — The sequence of primers used for quantitative RT-PCR (qRT-PCR) amplification and for methylation analysis. (DOC) [file pone.0102742.s003.doc]

**Table S1. The sequence of primers used for quantitative RT-PCR (qRT-PCR) amplification and for methylation analysis.**

| **Target** | **Primer** | **Sequence (5'-3')** | **Methods** |
| --- | --- | --- | --- |
| *PsbS1* mRNA | OsPsbS1_5' F | CTGTTCGGCAGGTCCAAGAC | qRT-PCR |
|  | OsPsbS1_5' R | CCGACGAACAGCTCGTTCTC | qRT-PCR |
| *Os03g0700800* mRNA | OsChIP_MgTF | CCTAGATCAGGGGTGTCTCC | qRT-PCR |
|  | OsChIP_MgTR2 | TCACATGCCTGAAAAATGCT | qRT-PCR |
| *OsActin 1* mRNA | OsChIP_AcF | GAGCTGTTATCGCCGTCCT | qRT-PCR |
|  | OsChIP_AcR | CAAGTGAGAACCACAGGTAGCA | qRT-PCR |
|  |  |  |  |
| Mitochondria genome | BSNB-mitF2 | GATAAGGAYGGATGTTGGGTGGGGTTG | primary PCR |
|  |  |  |  |
|  | BSNB-mitR1 | CTCACTACCTTTTTCAATCTTACAAC | primary PCR |
|  |  |  |  |
|  | BSNB-mitF3 | GGTGAGGAGGGAGTGTAGTGGG | nested PCR |
|  |  |  |  |
|  | BSNB-mitR2 | AAACATRCCTTCAACAACACCCTTA | nested PCR |
|  |  |  |  |
| *PsbS1* upstream region | BS-PsbF1 | TTGAGGAAAGAGAAGGYTGGAGAGGAAAGG | PCR for Sasanishiki |
|  |  |  |  |
|  | BS-PsbR1 | ACACCARCATCRACTRCRCCATCTCRCCCC | PCR for Sasanishiki |
|  |  |  | nested PCR for Habataki |
|  |  |  |  |
|  | BS-PsbF7 | TGAATYTGGTGGYTTGGGGGTAAATTG | primary PCR for Habataki |
|  |  |  |  |
|  | BS-PsbR2 | CATTCATCCATCCAAATTCCAACCTTTCTT | primary PCR for Habataki |
|  |  |  |  |
|  | BS-PsbF8 | ATGGAAYYGGTTGTTGYATGTGGT | nested PCR for Habataki |
