# Supplementary material for: Does the Upstream Region Possessing MULE-Like Sequence in Rice Upregulate PsbS1 Gene Expression?
Source: PLoS One. 2014 Sep 26;9(9):e102742. doi: 10.1371/journal.pone.0102742 (PMC4178011; doi:10.1371/journal.pone.0102742)
Supplement: Table S2 — Summary of characteristics of Os MULE elements in both japonica and indica rice cultivars. (DOC) [file pone.0102742.s004.doc]

**Table S2.** Summary of characteristics of *Os*MULE elements in both *japonica* and *indica* rice cultivars.

| Element^a^ | Total length (bp) | Lengths of TIRs (bp)^b^  Left / right | Target site duplication^c^  Left / right | Location | | | | | | |
| --- | --- | --- | --- | --- | --- | --- | --- | --- | --- | --- |
|  |  |  |  | Chromosome | | | Accession | | Position in the chromosome bp^d^ | |
| *Os*MULE-JSS1 | 2517 | 80/77 | cttttccag / cttttccag^f^ | | 1^e^ | [AP003286](http://www.ncbi.nlm.nih.gov/nucleotide/20160846?report=genbank&log$=nucltop&blast_rank=1&RID=UT9AK6Y601N) | | 37694124-37696640 | |  |
| *Os*MULE-JS2 | 136 | 78/72 | tctccaagttc / tctccaagttc^f^ | | 1 | AP003343 | | 30249273-30249408 | |  |
| *Os*MULE-JS3 | 139 | 78/70 | ctccggctc / ctccggctc^f^ | | 2 | AP005115 | | 33673076-33673214 | |  |
| *Os*MULE-JS4 | 136 | 78/72 | accggggaa / accggggaa^f^ | | 2 | AP005287 | | 32267864-32267999 | |  |
| *Os*MULE- JS5 | 136 | 78/72 | atgccaggc / atgccaggc^f^ | | 11 | AC120984 | | 23547301-23547436 | |  |
| *Os*MULE- IS6 | 171 | 78/70 | aacatgaaacat / aacatgaaaaga^g^ | | 1 | - | | 42920994-42921164 | |  |
| *Os*MULE- IS7 | 138 | 79/70 | catagcacc / catagcacc^f^ | | 1 | - | | 35235502-35235639 | |  |
| *Os*MULE-IS8 | 139 | 77/70 | atgttggat / atgttggat^f^ | | 2 | - | | 31771150-31771288 | |  |
| *Os*MULE-IS9 | 139 | 78/70 | cgacccgct / cgagccgct^f^ | | 5 | - | | 20934036-20934174 | |  |

^a^Mined elements are those obtained by homology searches using the most terminal 80-bp terminal inverted repeat (TIR) sequences of *japonica* specific sequence (JSS) as query sequence.

^b^5’ TIR sequences in the database were designated as left TIRs (and are shown to the left of the slash) in all elements, while 3’ TIRs were designated as right TIRs.

^c^Left and right target site duplications (TSDs) are separated by the slashes

^d^The positions of the terminal nucleotides of each *Os*MULE element within corresponding sequence accession are indicated

^e^JSS was found in the PAC clone [AP003286](http://www.ncbi.nlm.nih.gov/nucleotide/20160846?report=genbank&log$=nucltop&blast_rank=1&RID=UT9AK6Y601N) assigned to *japonica* rice chromosome 1.

^f^Perfect base pair of left and right TIR

^g^Imperfect base pair of left and right TIR by under line.
